# Beta‐adrenergic agonist induces unique transcriptomic signature in inguinal white adipose tissue

**DOI:** 10.14814/phy2.15646

**Published:** 2023-03-26

**Authors:** Henry A. Paz, Anna‐Claire Pilkington, Hannah D. Loy, Ying Zhong, Kartik Shankar, Umesh D. Wankhade

**Affiliations:** ^1^ Department of Pediatrics College of Medicine, University of Arkansas for Medical Sciences Little Rock Arkansas USA; ^2^ Arkansas Children's Nutrition Center Little Rock Arkansas USA; ^3^ Department of Pediatrics, Section of Nutrition University of Colorado School of Medicine, Anschutz Medical Campus Aurora Colorado USA

**Keywords:** beige adipose tissue, brown adipose tissue, CL316,243, thermogenesis, transcriptome, white adipose tissue

## Abstract

Activation of thermogenic adipose tissue depots has been linked to improved metabolism and weight loss. To study the molecular regulation of adipocyte thermogenesis, we performed RNA‐Seq on brown adipose tissue (BAT), gonadal white adipose tissue (gWAT), and inguinal white adipose tissue (iWAT) from mice treated with β3‐adrenoreceptor agonist CL316,243 (CL). Our analysis revealed diverse transcriptional profile and identified pathways in response to CL treatment. Differentially expressed genes (DEGs) in iWATCL were associated with the upregulation of pathways involved in cellular immune responses and with the upregulation of the browning program. We identified 39 DEGs in beige adipose which included certain heat shock proteins (Hspa1a and Hspa1b), and others suggesting potential associations with browning. Our results highlight transcriptional heterogeneity across adipose tissues and reveal genes specifically regulated in beige adipose, potentially aiding in identifying novel browning pathways.

## Graphical abstract

Activation of thermogenic adipose tissue depots has been linked to improved metabolism and weight loss. To study the molecular regulation of adipocyte thermogenesis, we performed RNA‐Seq on brown adipose tissue (BAT), gonadal white adipose tissue (gWAT), and inguinal white adipose tissue (iWAT) from mice treated with β3‐adrenoreceptor agonist CL316,243 (CL). Our results highlight transcriptional heterogeneity across adipose tissues and reveal genes specifically regulated in beige adipose, potentially aiding in identifying novel browning pathways.

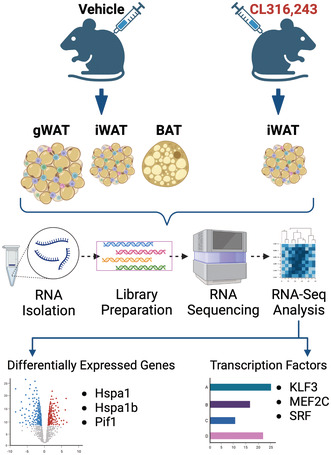

## 1 INTRODUCTION

Adipose tissue is an important indicator of the systemic energy status. Adipose tissue dysfunction during obesity and metabolic syndrome is well‐documented (Blüher, 2009). Of the forms of adipose depots, brown adipose tissue (BAT), which burns energy when active, is recognized as thermogenic tissue, and white adipose tissue (WAT) as a storage depot of lipids. In addition to major differences in their developmental origins, WAT is characterized by a large unilocular lipid droplet, whereas BAT has smaller, multilocular lipid droplets. Beige adipocytes are a type of fat cell that is capable of thermogenesis mimicking a BAT phenotype (Wu et al., 2012). Ucp1‐expressing, mitochondrial‐rich beige adipocytes are found in WAT depots of mice and develop in response to cold temperatures or pharmacological stimulation of β‐adrenergic signaling through agonists such as CL316,243 (CL) (Labbé et al., 2016; McMillan & White, 2015). Similar to BAT, beige adipocytes can generate heat via the mitochondrial protein Ucp1 that uncouples mitochondrial respiration from ATP synthesis. The discovery of metabolically active BAT in humans has led to increased interest in the thermogenic fat cell's potential role in the counteracting increased weight gain and related metabolic disorders such as type 2 diabetes, and metabolic syndrome (Yoneshiro et al., 2013).

Brown and beige adipose tissue are sites of adaptive thermogenesis in mice, and their activity contributes significantly to total energy expenditure. The previously held belief that adult humans do not have BAT was debunked in the last decade when three different groups demonstrated that adult humans have active BAT in response to environmental stimuli such as cold ambient temperature (Cypess et al., 2009; van Marken Lichtenbelt et al., 2009; Virtanen et al., 2009). Transcript profiling of active BAT depots in humans revealed that this thermogenic tissue express Ucp1, implying that certain human depots have a molecular profile similar to rodent BAT, while others have a beige fat‐like profile (Ikeda et al., 2018). Thermogenic fat activity in humans correlates with lean mass composition, indicating a possible role for brown and beige fat in adult human metabolism (Kiefer, 2017; van Marken Lichtenbelt et al., 2009). Pharmacological interventions can activate BAT and increase energy expenditure in humans. However, the relatively small volume of BAT may not contribute significantly to overall body fat reduction. Transforming WAT into BAT‐like or beige fat is a more conceivable possibility, as it has been shown to be possible in experimental animals. However, the evidence for transformation of WAT to beige/BAT‐like fat in humans is less solid.

Growing efforts of the scientific community are focused on unraveling the complexities of transcriptional regulation of brown and beige fat formation. Understanding cell‐specific development and function can be aided by defining each adipose tissue type's specific gene expression signature. This study sought to understand more about the transcript profiles of WAT, BAT, and beige adipose tissue in mice. We performed transcriptional profiling of various adipose tissue depots, namely inguinal WAT (iWAT), gonadal WAT (gWAT), and BAT. We used iWAT from CL treated mice as beige fat (iWATCL). RNA‐Seq permits the digital quantification of gene expression data, allowing for the assessment of relative abundance of genes within and between samples. We aim to identify genes that are specific to different adipose tissue types, especially beige, in order to improve our overall understanding of adipose tissue function and development. We demonstrate that the transcriptional profile of beige adipose tissue differs from that of WAT and BAT. Beige cells have a distinct transcriptional profile, which we believe is responsible, if not critical, for the browning process. An in‐depth examination of the transcriptional profile and the biological pathways that are associated with it provides comprehensive and unique information on beige cells.

## 2 MATERIALS AND METHODS

### 2.1 Experimental design

Male C57BL6/J mice were individually housed in an AAALAC‐approved animal facility in a temperature (22°C) and light controlled room (12 h light‐12 h dark cycle). To avoid the potential distortion of the baseline for measuring transcriptional changes related to adipose thermogenesis, mice were acclimated to the housing conditions for several weeks prior to the start of the experiment. Additionally, all mice were subjected to the same housing conditions throughout the experiment, which allowed us to compare the differential response. The Institutional Animal Care and Use Committee at the University of Arkansas for Medical Sciences approved all experimental protocols. Starting at 5 weeks of age, mice were given ad libitum access to control diet (17% fat Harlan Teklad, TD95095) for 15 weeks. At 20 weeks of age, mice were treated with a daily intraperitoneal injection of β3‐adrenergic agonist CL (*n* = 5) (1 mg/kg body weight) or vehicle (saline solution; *n* = 5) for 7 days. On the morning of the 8th day, mice were euthanized by carbon dioxide asphyxiation and the interscapular BAT, gWAT, and iWAT were dissected carefully to avoid contamination with adjacent tissues such as muscle, weighed and fixed in 10% formalin to perform histological examination. Pieces of tissues were flash‐frozen in liquid nitrogen and stored at −80°C until further analysis. For histomorphometric analyses 3–4 mm pieces of adipose tissue from the inguinal fat depot were fixed in buffered alcoholic formalin for 4 days and embedded in paraffin using routine histological procedures. Sections (6 μm thick) were stained with hematoxylin and eosin.

### 2.2 RNA extraction and RNA‐Seq library preparation

Total RNA was isolated from 50 mg of adipose tissue using a combination of TRI reagent and RNeasy‐mini columns (Qiagen), including on‐column DNase digestion. RNA quality and integrity was confirmed spectrophotometrically (A260/A280 ratio > 1.9) and via visualization using Experion RNA Std‐Sens chips (BioRad). Equal amounts of total RNA from 1–2 mice were pooled, to generate three biologically distinct replicates per group representing all animals (*n* = 5). Poly‐A RNA was isolated from 5 μg of total RNA using Dynabeads® mRNA‐Direct kit (Invitrogen) and procedures described previously (Wankhade et al., 2017). Briefly, poly‐A RNA was captured by addition of 100 μL of Oligo‐(dt)_25_ Dynabeads in 150 μL of lysis buffer. The mixture was incubated on a rotary shaker for 20 min at room temperature. mRNA‐bead complexes were washed twice with 100 μL of wash buffer A (10 mM Tris–HCl, pH 7.5, 0.15 M LiCl, 1 mM EDTA, 0.1% LiDS), followed by two washes (100 μL each) with wash buffer B (10 mM Tris–HCl, pH 7.5, 0.15 M LiCl, 1 mM EDTA). RNA was eluted from the beads in 11 μL of nuclease free water by heating to 65°C for 5 min. Stranded mRNA‐Seq library construction was carried out using NEB‐Next Ultra reagents (New England Biolabs). First and second strand cDNA synthesis, end‐filling using Klenow fragment, and dA‐tailing were carried out using manufacturer's recommendations. Ligation with Illumina's paired‐end adapters for multiplexed sequencing was performed with 1 μL of T4 DNA ligase, 0.3 μM of annealed adapters, in a 50 μL reaction volume for 30 min at room temperature. Ligated products were separated using a high‐resolution 2% agarose gel, and products around 200 bp (±50 bp) were excised and purified using Qiagen gel extraction kit (Qiagen). Size‐selected cDNA libraries were amplified using indexed primers. PCR was carried out for 12–14 cycles using 29 μL of template, 1 μL of forward and reverse primers (25 μM), and 1 U Phusion high‐fidelity DNA polymerase (New England Biolabs). PCR products were purified using Qiaquick PCR purification columns (Qiagen) and eluted in 30 μL final volume. A small aliquot (~1 μL) was evaluated using DNA1K chip (Experion, Bio‐Rad) to confirm the absence of primer‐dimers and other spurious products. Quantification of the RNA‐seq libraries was done via Qubit dsDNA HS Assay kit.

### 2.3 RNA‐Seq analysis

High‐quality reads were mapped against the mouse reference genome (GRCm38_v100) using TopHat (Trapnell et al., 2009) and resulting BAM files were used in SeqMonk v1.47.2 for transcript quantitation. Raw counts were normalized to log_2_ RPM values and only genes with log_2_ RPM > 0 were considered for analysis to minimize biological noise. Filtered data were transformed back to raw counts and differentially expressed genes (DEGs) were detected using the DESeq2 algorithm (Love et al., 2014) with gWAT from vehicle treated group as the control. A gene was considered differentially expressed when the false discovery rate (FDR) corrected *p*‐value was ≤0.05 and the absolute value of the log_2_ fold change was ≥2.

### 2.4 Gene ontology analysis

The list of DEGs were analyzed using the Ingenuity Pathway Analysis (IPA, version 73,620,684) core analysis (Krämer et al., 2014). IPA uses the right‐tailed Fisher's Exact test to calculate significance where the *p*‐value is the probability of overlap between the treatment gene set and the IPA Ingenuity Knowledge Base reference gene set. To predict pathway activity, IPA uses a separate Z‐score test. To determine transcription regulators, the Upstream Analysis from IPA was used. For Canonical Pathway, Diseases and Functions, and Upstream Analyses, the significance threshold was considered at ‐log(*p*‐value) ≥ 1.3 and significant inhibition or activation were defined at Z‐score ≤ −2 or Z‐score ≥ 2, respectively. Analyses of pathway enrichment from the distinctive upregulated or downregulated gene sets of iWATCL were done using EnrichR (Kuleshov et al., 2016).

### 2.5 Statistical analysis and data representation

Unconstrained ordination was computed, and figures were generated using R v4.2.0 (R Core Team, 2022). A principal coordinate analysis (PCoA) using Bray‐Curtis dissimilarities was conducted and differences in transcriptome profile among samples were visualized in a two‐dimensional PCoA plot. To further evaluate transcriptome profiles, a dendrogram was constructed using Euclidean distances and the Ward's method for hierarchical clustering. The profiling of fat tissue types (ProFAT) pipeline (http://ido.helmholtz‐muenchen.de/profat/) was used to determine the browning capacity among samples (Cheng et al., 2018). This computational tool uses robust gene signatures from white and brown adipocytes to predict thermogenic potential. Volcano plots were used to show the number of upregulated and downregulated DEGs from each adipose depot. Venn diagrams were used to identify distinctive DEGs within each adipose tissue or those shared among adipose tissues. Interactome networks were generated using the STRING database (Search Tool for Retrieval of Interacting Genes/Proteins) (Szklarczyk et al., 2019) where nodes are proteins and edges represent predicted functional associations.

## 3 RESULTS

### 3.1 Adipose tissue transcriptome clustered together based on adipose tissue type

**FIGURE 1:**
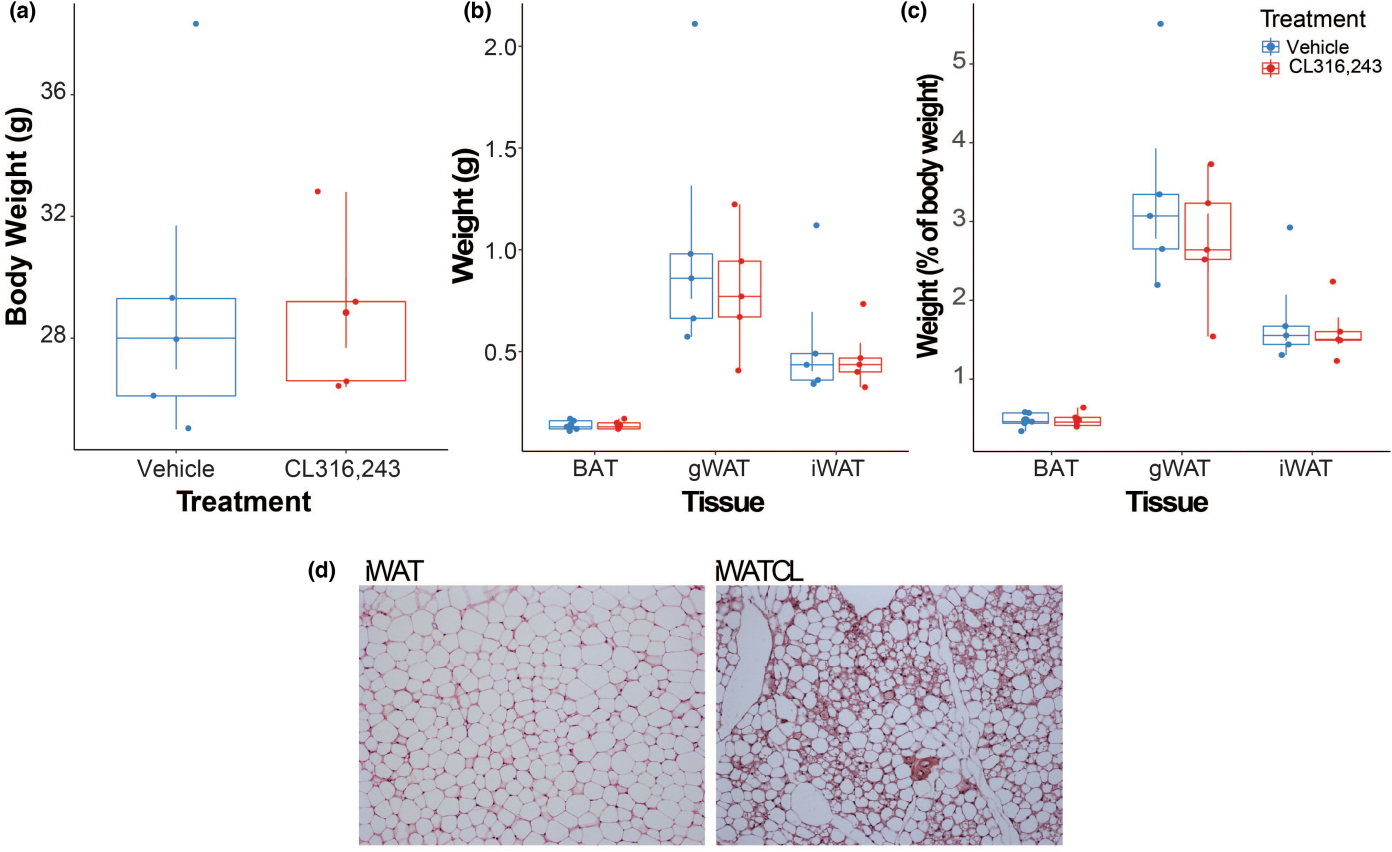
Body and adipose depot weights from vehicle‐ and CL316,243‐treated mice. (a) Body weights, (b) adipose depot weights, (c) adipose depot weights relative to body weight and (d) hematoxylin and eosin (H&E) stained sections of adipose depots.

**FIGURE 2:**
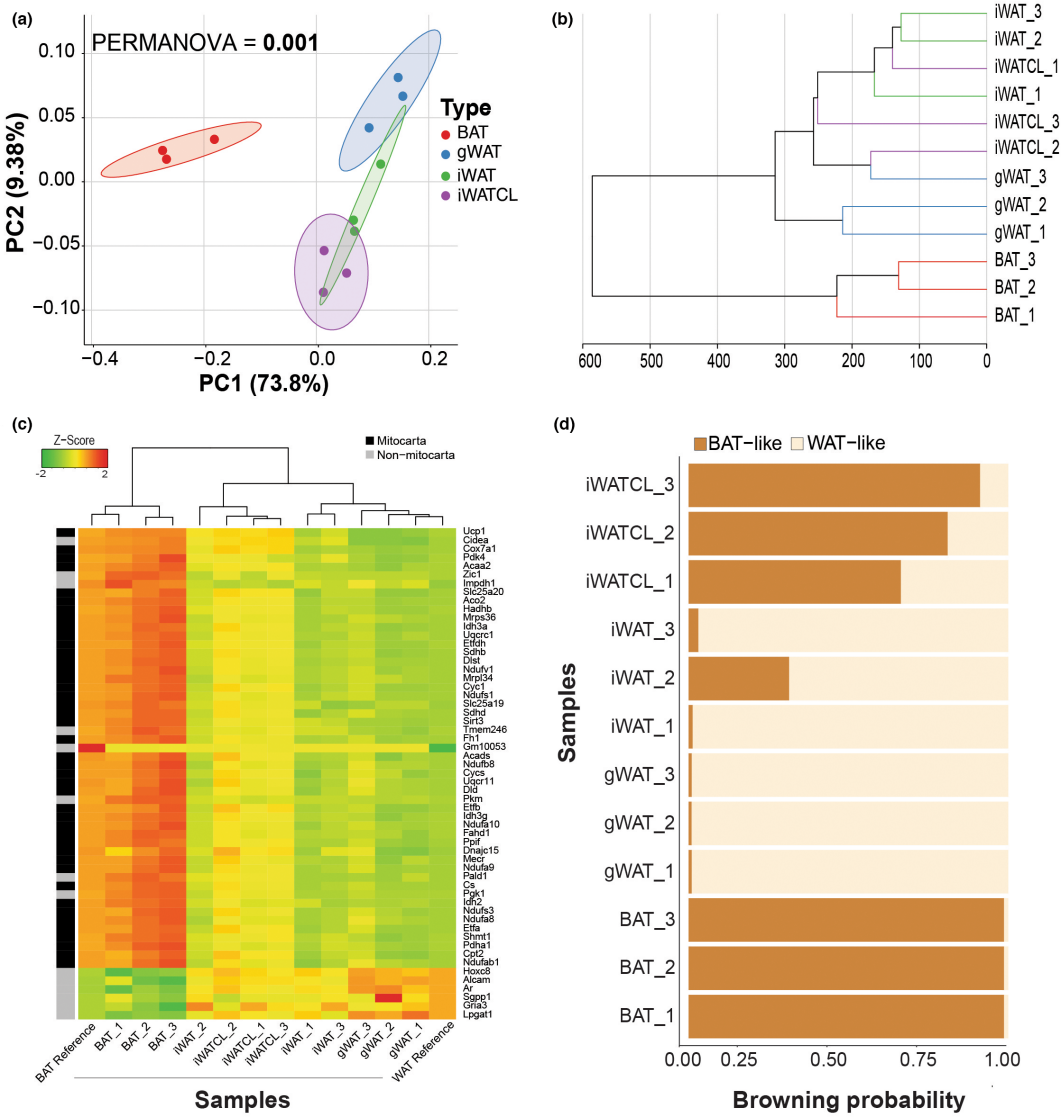
Transcriptome profile and browning potential of different adipose tissue depots. Clustering of samples by adipose tissue depot shown in a (a) two‐dimensional principal coordinate analysis (PCoA) plot based on Bray‐Curtis dissimilarities and in a (b) dendrogram constructed using Euclidean distances and the Ward's method for hierarchical clustering. (c) Heatmap showing the relative gene expression of marker genes from brown and white adipose tissues and (d) predicted browning capacity for each sample based on the profiling of fat tissue types (ProFAT) pipeline.

A week‐long stimulation of β‐adrenergic pathway via CL did not induce weight changes in mice in comparison to vehicle‐treated mice. (Figure 1a). Likewise, weights of adipose tissues were similar between treatments (Figure 1b,c). As expected, treatment with CL promoted the appearance of multilocular brown‐like adipocytes in iWAT (Figure 1d). RNA transcriptome analysis of gWAT, iWAT, BAT (vehicle‐treated mice), and iWATCL (CL‐treated mice), included ~396 million reads (~33 million/sample) covering 12 biological replicates. Alignment of high‐quality reads showed that mapping to exons ranged from 91.2% to 94.5% across samples with no reads mapping to mitochondrial or ribosomal RNA (Table S1). To assess the global transcriptome profile of adipose tissues, a PCoA was conducted including all annotated genes. We found distinct profiles in adipose tissue depots (PERMANOVA = 0.001) based on type. In the PCoA plot (Figure 2a), samples clustered by location, with WAT depot samples being more similar to each other than BAT samples. The results of further analysis of the transcriptomes using hierarchical clustering (Figure 2b) were consistent with the PCoA. Transcriptome‐based meta‐analyses recently discovered distinct molecular signatures between brown and white adipocyte phenotypes and identified particular, basic marker‐genes for phenotype prediction. Using gene‐expression data from these markers, adipocyte thermogenic capacity could be estimated (Kuleshov et al., 2016). Analysis using ProFAT showed that gene expression levels of BAT marker‐genes were higher in iWATCL than in iWAT and gWAT, as expected (Figure 2c). Accordingly, iWAT from CL‐treated mice had a greater browning capacity than iWAT and gWAT from vehicle‐treated mice (Figure 2d).

### 3.2 Browning and immune‐related pathways are increased during β‐adrenergic stimulation in iWAT

**FIGURE 3:**
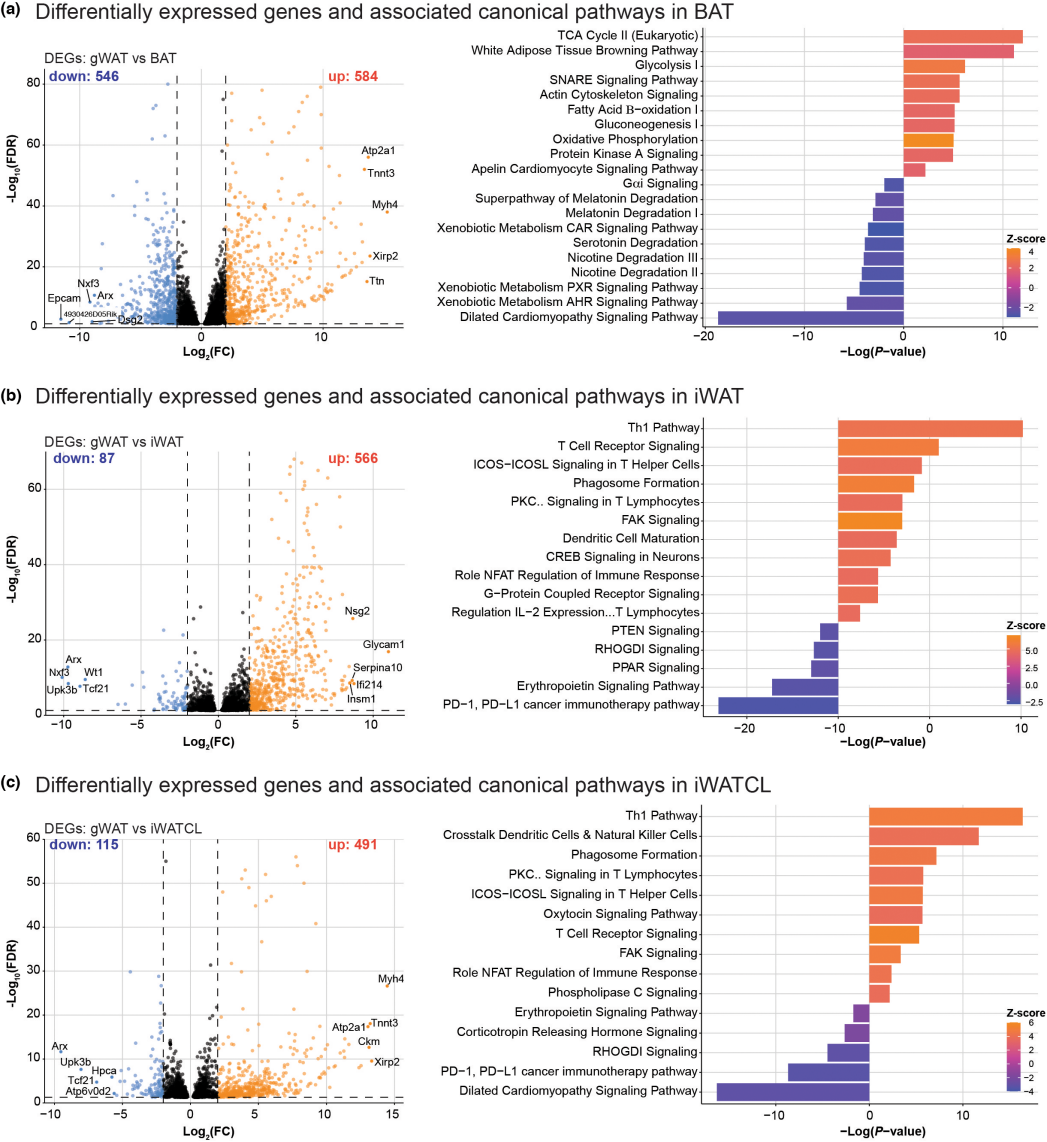
Differentially expressed genes and associated canonical pathways among adipose depots. Blue, black and orange dots in volcano plots represent downregulated, not significant and upregulated genes; respectively, in (a) BAT, (b) iWAT, and (c) iWATCL. DEGs were considered when FDR adjusted *p*‐value ≤ 0.05 and log2 |fold change| ≥ 2. Divergent bar charts displaying significant enriched canonical pathways where inhibition or activation were defined at Z‐score ≤ −2 or Z‐score ≥ 2, respectively.

Genes with log2 RPM values >0 which amounted to nearly 41% of total identified transcripts were considered for further analysis. We identified DEGs in BAT, iWAT, and iWATCL using gWAT from vehicle‐treated group as the control. The number of DEGs were greater in BAT (1130) followed by iWAT (653) and iWATCL (606) (Figure 3). Evaluation of the top five upregulated and downregulated DEGs (i.e., genes exhibiting greater fold change) showed that iWATCL shared more top upregulated genes with BAT (Atp2a1, Myh4, Tnnt3, and Xirp2) and more top downregulated genes with iWAT (Arx, Tcf21, and Upk3b). DEGs were then subjected to IPA to identify significantly enriched canonical pathways. Complete results of the core analysis among adipose tissues are presented in Table S2. Canonical pathways were sorted by *p*‐value and Z‐score and the top 10 significantly upregulated and downregulated pathways were identified (Figure 3). In BAT, top activated pathways included the browning pathway and pathways involved in the generation of energy such as the TCA cycle, glycolysis and oxidative phosphorylation, whereas top downregulated pathways were involved in degradation, and xenobiotic metabolism (Figure 3a). In iWAT, the top activated pathways included pathways involved in cellular immune responses such as the Th1 pathway and T cell receptor signaling, while PPAR signaling was among the top downregulated pathways (Figure 3b). The profile of top activated pathways in iWATCL was similar to iWAT; however, like BAT, the browning pathway was significantly activated (Table S2) and the dilated cardiomyopathy signaling pathway was among the top downregulated pathways (Figure 3c).

Further assessment of biological processes associated with DEGs among adipose tissues was done using the diseases and functions analysis from IPA. Annotations were sorted by *p*‐value and Z‐score and the top 10 significantly upregulated and downregulated functions are shown in Figure S1. In BAT, thermogenesis, energy homeostasis and muscular associated functions were activated, while functions related to muscular disorders were downregulated. Consistent with the canonical pathway analysis, iWAT and iWATCL had a similar profile of upregulated functions which were mainly related to the general category of lymphoid tissue structure and development. The profiles of downregulated functions for iWAT and iWATCL were mainly related to immune signaling pathways.

### 3.3 Heat shock proteins are downregulated in iWATCL

**FIGURE 4:**
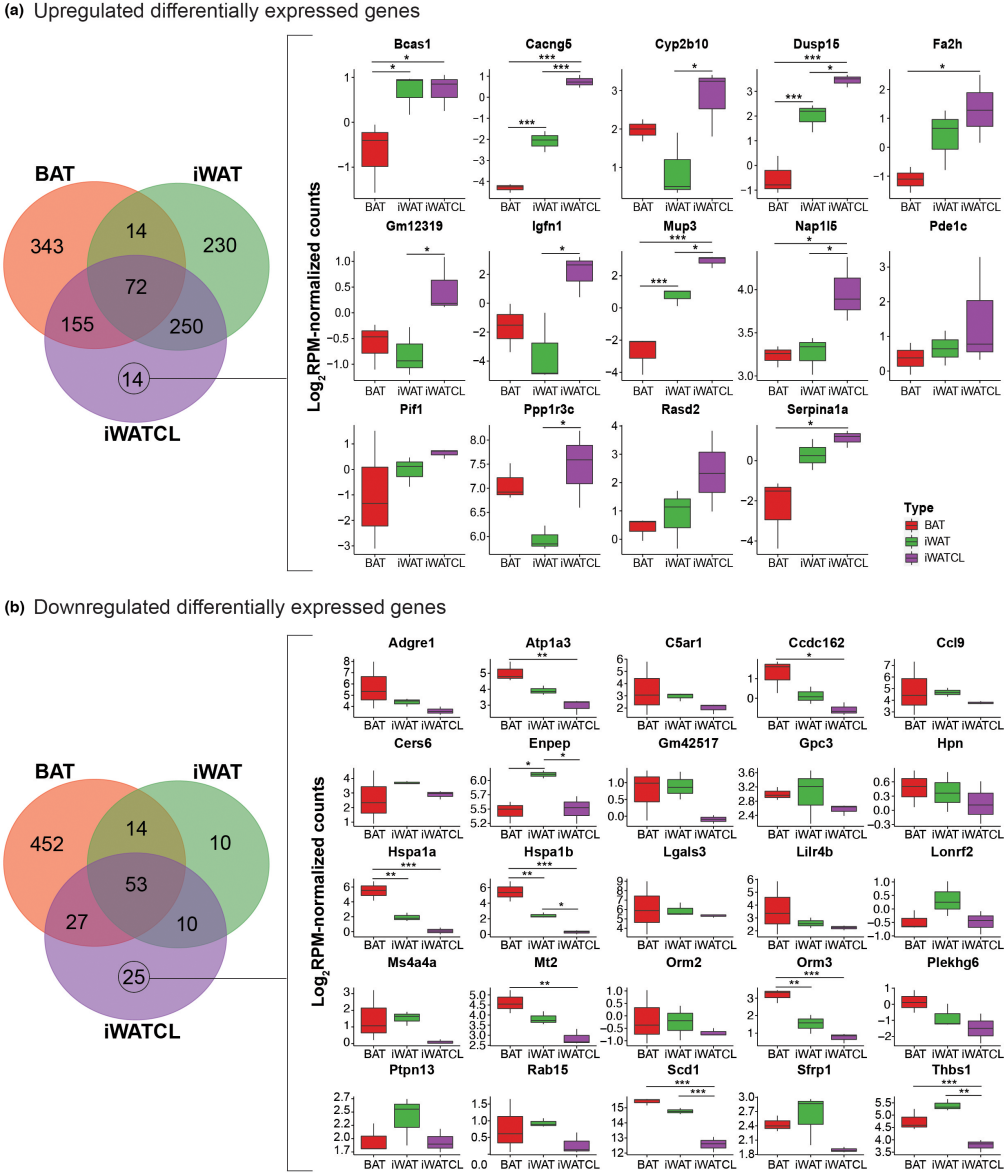
Distinctive and shared differentially expressed genes among adipose depots. (a) Upregulated and (b) downregulated DEGs and expression levels of unique DEGs in iWATCL. *p < 0.05, **p < 0.01, ***p < 0.001, one‐way ANOVA with Tukey honest significant differences (HSD) post hoc test.

**FIGURE 5:**
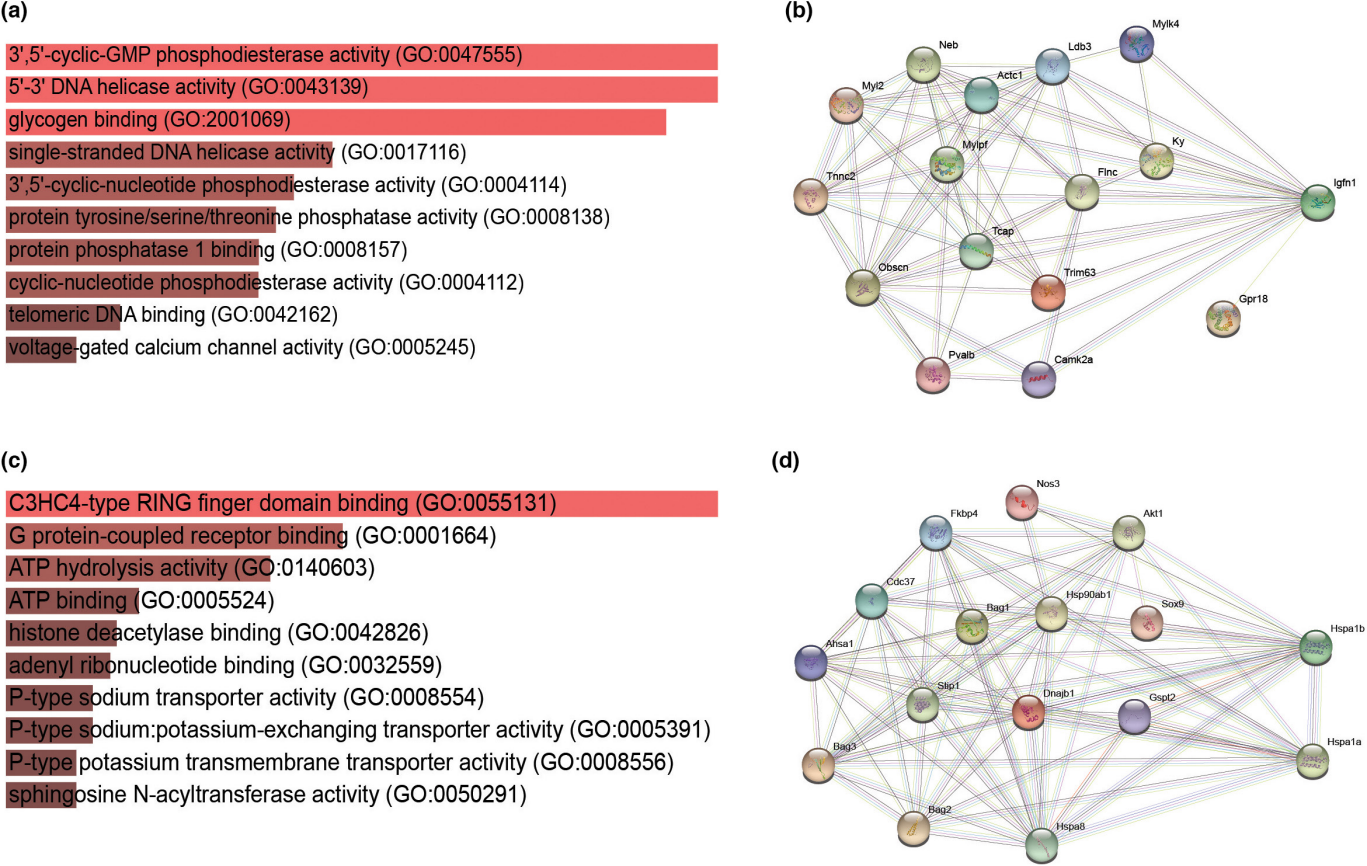
Functions associated with distinctive differentially expressed genes in iWAT from mice stimulated with CL316,243. Enriched molecular functions and interaction network of upregulated genes (a, b) and of downregulated genes (c, d).

Identification of DEGs that were distinctive to each adipose depot was performed using Venn diagrams (Figure 4). Distinctive DEGs in iWATCL were of particular interest as these genes could represent potential genetic markers and could also aid to identify molecular mechanisms that differentiate this adipose tissue. For upregulated DEGs, the Venn diagram indicated that 343, 230 and 14 genes were selective to BAT, iWAT, and iWATCL, respectively (Figure 4a). For DEGS distinctive to iWATCL, evaluation of the expression levels showed that *Cacng5*, *Dusp15*, *Mup3* and *Nap1l5* were greater (*p* < 0.05) in iWATCL compared to both BAT and iWAT. The expression level of other DEGs such as *Bcas1*, *Fa2h*, and *Serpina1a* was greater in iWATCL compared to BAT but similar to iWAT. Distinctive genes in iWATCL were associated with molecular functions such as glycogen binding and voltage‐gated calcium channel activity (Figure 5a). Interactome analysis revealed that *Igfn1* had the highest number of interactions with other upregulated DEGs in iWATCL and was associated with a muscle contraction regulation network (Figure 5b). For downregulate DEGs, the Venn diagram indicated that 452, 10 and 25 genes were selective to BAT, iWAT, and iWATCL, respectively (Figure 4b). For downregulated DEGS distinctive to iWATCL, expression levels of *Atp1a3*, *Ccdc162*, *Hspa1a*, *Hspa1b*, *Mt2*, *Orm3*, *Scd1*, and *Thbs1* were lower (*p* < 0.05) in iWATCL compared to BAT. The expression levels for *Hspa1b*, *Scd1* and *Thbs1* were also lower (*p* < 0.05) in iWATCL compared to iWAT. These distinctive downregulated DEGs were associated with functions of ATPase activity such as ATP binding and hydrolysis (Figure 5c). Interactome analysis showed *Hspa1a* and *Hspa1b* being important genes in a protein folding chaperone network (Figure 5d).

### 3.4 Potential transcription factors that regulate browning in iWAT

**FIGURE 6:**
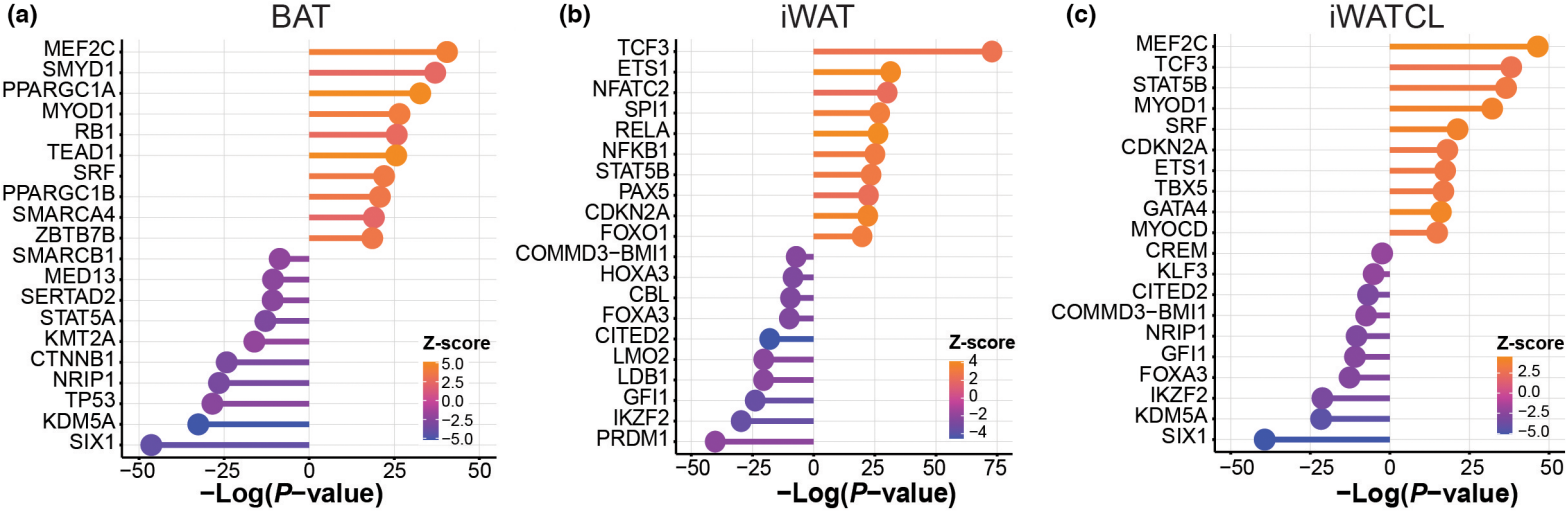
Top 10 upregulated and downregulated transcription regulators associated with differentially expressed genes among adipose depots. Inhibition or activation were defined at Z‐score ≤ −2 or Z‐score ≥ 2, respectively.

Transcription factors are proteins that coordinate gene expression in specific cell types in a spatial and temporal manner. These proteins play a role in transcriptional regulation, influencing the expression of key genes involved in critical physiological functions. The Upstream Analysis from IPA was used to determine transcription factors associated with DEGs impacted by CL stimulation (Figure 6). From the top activated transcription factors, iWATCL shared two transcription factors with BAT (*MEF2C* and *MYOD1*) and none with iWAT. This suggests differences in the processes regulating browning in iWAT compared to BAT. Activated transcription factors in iWATCL were mainly immune (*TCF3*, STAT5B, *ETS1*) or muscle related (*TBX5*, *GATA4*, *MYOCD*). Whereas from the top downregulated transcription factors, iWATCL shared three with BAT (*KDM5A*, *NRIPL*, and *SIX1*) and three with iWAT (*COMMD3‐BMI1*, *FOXA3*, and *GFI1*).

## 4 DISCUSSION

The study investigated the effects of β3‐adrenergic stimulation via CL on the browning of white adipose tissue in mice. The largest mouse adipose‐centric gene expression atlas, ProFAT, was used to assess browning potential and it was found that iWAT had the most potential for beiging. Differentially expressed genes in iWATCL included heat shock proteins (Hsp), Pif1, Ppp1r3c, Cyp2b10, Hpn, Sfrp1, and Thbs1. Hsp were found to have an association with the browning process in iWAT, with some Hsp being upregulated in BAT at 22°C while others were downregulated in iWAT after CL stimulation. Transcription factors such as KLF3, MEF2C, and MYOD1 were also found to be differentially expressed in iWATCL, potentially playing a role in the browning of WAT. The results suggest a potential link between Hsp and the browning process and the involvement of several transcription factors in the development of beige adipose tissue.

β‐3‐adrenergic stimulation with CL modulates the concentrations of metabolites related to glucose metabolism in fat depots along with emergence of beige adipocytes in iWAT and increased inflammatory markers in gWAT (Fujimoto et al., 2019; Granneman et al., 2005). In our study, DEGs in iWAT and gWAT showed upregulation of inflammatory pathways along with increased BAT specific genes in response to CL in iWAT. One of the upregulated genes in beige tissue was *Pif1*, which was previously shown to be critical player in metabolism, lack of which drove weight gain and decreased the exercise drive in mice (Belmonte et al., 2019). Other upregulated genes included *Ppp1r3c* and *Cyp2b10*. *Ppp1r3c* is enriched in adipocytes and is noteworthy that it increases during the browning process (Keinan et al., 2021) and *Cyp2b10* is known to be regulated by nuclear receptors like PXR, repression of which leads to obesity and exacerbation of metabolic disease by disrupting metabolism of fatty acids (Chen et al., 2021). Among the downregulated transcripts in beige tissue, *Hpn* (hepsin) was previously reported to enhance liver metabolism and inhibit adipocyte browning in mice (Li et al., 2020; Li et al., 2021). Hepsin‐deficient mice are resistant to obesity, hyperglycemia, and hyperlipidemia (Li et al., 2020). Another downregulated gene, *Sfrp1* is known to express in mature adipocytes and modulates the paracrine regulation of adipogenesis via Wnt/β‐catenin signaling (Lagathu et al., 2010). Thbs1 was also downregulated in our dataset, and it has previously been reported to express highly in visceral adipose tissue, loss of which makes mice resistant to diet‐induced weight gain (Inoue et al., 2013). In addition, Thbs1 is elevated in insulin resistant and obese humans (Varma et al., 2008). The differentially expressed genes observed in CL‐treated iWAT are likely derived from beige cells, which are formed from white adipocytes through recruitment or transformation. The comparison to gWAT as a control led to the identification of DEGs. Our interpretation is supported by the current literature and single‐cell RNA sequencing studies on various adipose tissue cell types. Specifically, we propose that the observed changes in genetic signature may contribute to the transformation of white adipocytes into beige cells (Burl et al., 2018; Ramirez et al., 2020).

Heat shock proteins are produced in response to multiple stressors and conduct chaperone roles such as protein folding (Park & Seo, 2015). Hsp are commonly named based on their molecular size. Emerging evidence suggests a greater involvement of Hsp on browning of adipose tissue. One study reported that in Hsp20 knockout mice, the thermogenic capacity of WAT increased compared to the wild type mice and that this response was mediated through the regulation of PPARγ (Peng et al., 2018). Another study showed that the deficiency of Hspa12a, member of the Hsp70 family, promoted greater browning in iWAT of knockout mice compared to wild type during cold exposure and suggested this effect could be mediated through paracrine mechanisms (Cheng et al., 2019). In contrast to the above, Kim et al found that *Hsph1*, *Hsp90aa1*, and *Hspa8* are upregulated in BAT of young and old mice exposed to acute cold exposure (Kim et al., 2021). In the current study, the expression of *Hspa1a* and *Hspa1b* were lower in iWAT compared to BAT and β3‐adrenergic stimulation through CL further decreased their expression. These results although not conclusive, suggest associations between Hsp and the browning process in WAT. Additionally, some of the non‐discussed genes here (differentially and uniquely expressed in CL treated iWAT) are mainly implicated in nervous system‐related process, while they are not reported to be involved in adipose tissue‐related processes, it could be a potential area of exploration.

Adipocyte development is a highly orchestrated process that can vary between different fat depots. To date, many transcription factors have been found to play important roles in this development by binding to key genes and influencing their expression. In the current dataset, when differentially expressed genes were probed for their transcription factor associations, several interesting targets were revealed. KLF3, MEF2C, MYOD1, SRF, KDM5A, TCF3, and FOXA3 are some of the transcription factors that were differentially expressed in the data. KLF3, for example, has been established to play a role in adipogenesis and KLF3 knockout mice were found to have fewer adipogenic cells, indicating that it may have a key role in beige adipose tissue development (Sue et al., 2008). MEF2C, on the other hand, stimulates miR222 that inhibits SCD5 and thus decreases fat deposition. MYOD1 interacts with glucocorticoid receptors to regulate the size of myofibers and may have a role in the browning process (Ren et al., 2020) and it was upregulated in iWATCL in our dataset. SRF regulates MRTF‐A and regulates adipogenesis via actin dynamics (Mikkelsen et al., 2010), while KDM5A may play a role in adipocyte differentiation. KDM5A is a histone demethylase that is a transcriptional corepressor and that was downregulated in both BAT and iWATCL. Research shows that when KDM5A expression is decreased, the Wnt/beta‐catenin pathway that leads to preadipocyte differentiation is also decreased (Guo et al., 2019). TCF3 may be essential for white adipose development (Guo et al., 2012), while FOXA3 inhibits PGC1α and CREB binding (Ma et al., 2014), which could indicate its role in metabolic pathway activation and the control of thermogenesis. These findings could provide valuable future research targets.

The present study provides a detailed transcriptome profile of various adipose tissues, including beige adipose tissue. However, it is crucial to acknowledge that whole adipose tissue contains multiple cell types, such as preadipocytes, stromal vascular cells, immune cells, and adipocytes, among others, and therefore, caution should be exercised when interpreting the transcriptome readout from whole tissue. In this study, CL was used as a browning stimulant, although other factors such as cold ambient temperatures, exercise, and pharmacological agents can also induce browning and may result in different transcriptomic profiles of beige adipose tissue. Our study employed C57B6 mice, which are a commonly used strain for metabolic studies. However, it should be noted that the metabolic profile of other strains may differ from that of C57B6 mice. For example, SV129 mice are known to be more resistant to weight gain on a high‐fat diet due to their higher amount of brown adipose tissue, while C57B6JN mice have a mitochondrial metabolism impairment due to a Nicotinamide nucleotide transhydrogenase gene mutation (Ferrannini et al., 2016; Nicholson et al., 2010). Therefore, our findings may not be generalizable to other strains with different metabolic profiles.

In conclusion, our genome‐wide transcriptome analysis of various adipose tissue depots reveals that beige adipose tissue has a distinct set of genes and a unique transcriptional profile compared to WAT and BAT. β3‐adrenergic receptor agonist leads to differential regulation of specific genes in beige adipose tissue, with many of the differentially regulated genes being linked to metabolism and obesity. These findings deepen our understanding of the adipose tissue transcriptome and could lead to the discovery of novel pathways that control adipose tissue physiology and may be disrupted in obesity. Overall, this study opens new avenues for research into the functions of genes and transcriptional factors in adipose tissue development and pathology.

### AUTHOR CONTRIBUTIONs

Umesh D. Wankhade conceptualized the study; Umesh D. Wankhade, Kartik Shankar, Henry A. Paz, Y.Z., Anna‐Claire Pilkington, and Hannah D. Loy conducted the experiments; Henry A. Paz, Umesh D. Wankhade, and Kartik Shankar performed the data analysis; Henry A. Paz, Umesh D. Wankhade, Anna‐Claire Pilkington, and Hannah D. Loy wrote the manuscript. All authors have read and agreed to the published version of the manuscript.

### FUNDING INFORMATION

This research was funded in part by the United States Department of Agriculture‐Agricultural Research Service Project 6026–51000‐010‐05S and National Institute of Diabetes and Digestive and Kidney Diseases Grant R01‐DK‐084225 (to K. Shankar). K.S. is supported in part by grants from the NIH (5 P30DK048520–27 and 1 R01HD102726‐01A1) and funds from the Department of Pediatrics, University of Colorado Anschutz Medical Campus and the Anschutz Health and Wellness Center. U.W. is also supported by the Arkansas Children's Research Institute, the Arkansas Biosciences Institute, and the Center for Childhood Obesity Prevention funded under the National Institutes of Health (P20GM109096). Research reported in this publication was supported by the National Center For Advancing Translational Sciences of the National Institutes of Health under award number UL1 TR003107. The content is solely the responsibility of the authors and does not necessarily represent the official views of the National Institutes of Health.

### CONFLICT OF INTEREST STATEMENT

The authors declare no conflict of interest.

### ETHICS STATEMENT

The reported research here was conducted in accordance with the guidelines of the Institutional Animal Care and Use Committee (IACUC) and the National Institutes of Health (NIH) Guide for the Care and Use of Laboratory Animals. The experimental procedures were designed to minimize animal suffering and ensure the welfare of the animals used in this study. This is authors' own original work, which has not been previously published elsewhere. The paper reflects the authors' own research and analysis in a truthful and complete manner.

## Supporting information


Figure S1.

Table S1.

Table S2.
