# Supplementary figures and images for: Beta‐adrenergic agonist induces unique transcriptomic signature in inguinal white adipose tissue
Source: Physiol Rep. 2023 Mar 26;11(6):e15646. doi: 10.14814/phy2.15646 (PMC10040403; doi:10.14814/phy2.15646)

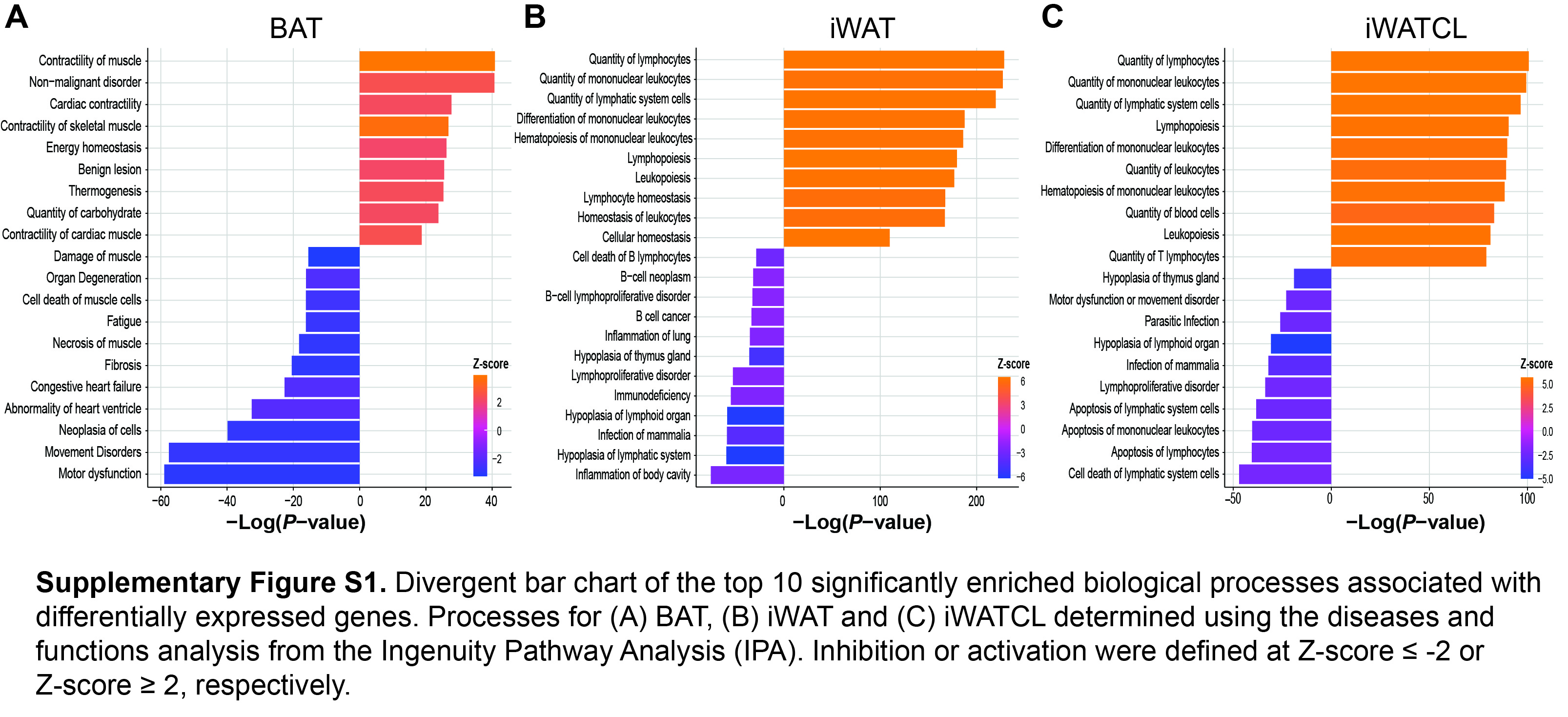

Supplement: Supplementary file 1 — Figure S1. [file PHY2-11-e15646-s002.jpg]
